# Endogenous microparticles drive the proinflammatory host immune response in severely injured trauma patients

**DOI:** 10.1186/cc14393

**Published:** 2015-03-16

**Authors:** N Curry, K Balvers, DJ Kleinveld, AN Boïng, R Nieuwland, JC Goslings, NP Juffermans

**Affiliations:** 1 John Radcliffe Hospital, Oxford, UK; 2 Academic Medical Center, Amsterdam, the Netherlands

## Introduction

Severe trauma affects the immune system, which in its turn is associated with poor outcome. The mediators driving the immune responses in trauma are largely unknown. The aim of this study was to investigate the role of endogenous microparticles (MPs) in mediating the immune response following severe trauma.

## Methods

A prospective, observational substudy of the Acute Coagulopathy and Inflammation in Trauma (ACIT) II study was performed at our academic level 1 trauma center. Adult multiple trauma patients with an Injury Severity Score of 15 or higher were included between May 2012 and June 2013. *Ex vivo *whole blood stimulation with lipopolysaccharide was performed on aseptically collected patient plasma containing MPs and in plasma depleted of MPs. Flow cytometry and transmission electronic microscopy were performed on plasma samples to investigate the numbers and cellular origin of MPs. Healthy individuals served as a control group.

## Results

Ten trauma patients and 10 healthy individuals were included. Trauma patients were significantly injured with a median ISS of 19 (17 to 45). On admission to the hospital, the host response to bacterial stimulation was blunted in trauma patients compared with healthy individuals, as reflected by decreased production of IL-6, IL-10 and TNFα (*P *< 0.001). In trauma patients, MP-positive plasma was associated with a significantly higher synthesis of IL-6 and TNFα compared with plasma depleted from MPs (*P *= 0.047 and 0.002 respectively). Compared with healthy individuals the number of circulating MPs was significantly decreased in trauma patients (*P *= 0.009). Most MPs originated from platelets. Multiple cellular protrusions, which result in MP formation, were observed in plasma from trauma patients, but not in healthy controls.

## Conclusion

On admission, trauma patients have a reduced immune response towards endotoxin challenge which is, at least in part, mediated by MPs, which circulate in low numbers and in early stages. Most MPs originate from platelets, which indicates that these cells may be the most important source of MPs involved in initiating an inflammatory host response post injury.

